# Karyomegalic interstitial nephritis: A case series and review of the literature on genetic insights and clinical challenges 

**DOI:** 10.5414/CNCS111727

**Published:** 2025-06-02

**Authors:** Seyda Gul Ozcan, Durdane Yagmur Ersoy, Ali Osman Polat, Iclal Gurses, Aysel Kalaycı Yigin, Sinan Trabulus, Nurhan Seyahi

**Affiliations:** 1 Department of Nephrology,; 2 Department of Internal Medicine,; 3 Department of Pathology, and; 4 Department of Medical Genetics, Istanbul University – Cerrahpasa, Cerrahpasa Medical Faculty, Fatih, Istanbul, Türkiye

**Keywords:** karyomegalic interstitial nephritis, nephritis, interstitial, chronic kidney disease

## Abstract

Karyomegalic interstitial nephritis (KIN) is a rare hereditary form of chronic interstitial nephritis that was first described over 50 years ago. It is characterized by karyomegalic tubular epithelial cells and progressive chronic kidney disease, often leading to end-stage renal disease by the fifth decade of life. Recent studies have identified FAN1 mutations as a key genetic contributor, with additional associations to environmental factors and toxic exposures, such as ochratoxin A, alkylating agents, and heavy metals, which may act as potential triggers of the disease. We present a detailed analysis of KIN cases, highlighting genetic diversity, clinical manifestations, and management challenges, complemented by a comprehensive review of the literature.

## Introduction 

Karyomegalic interstitial nephritis (KIN) is a rare hereditary form of chronic interstitial nephritis that was first described ~ 50 years ago [1]. The term “KIN” was introduced by Mihatsch et al. [2], who reported 3 cases of systemic karyomegaly associated with chronic interstitial nephritis in 1979. The disease typically presents as a slowly progressive form of chronic kidney disease, often leading to end-stage renal disease before the age of 50 [3]. Despite growing recognition, KIN remains an underdiagnosed cause of interstitial nephritis. Extrarenal manifestations, such as recurrent upper respiratory tract infections and abnormal liver function tests, are generally mild or absent [4]. The hallmark of KIN is the presence of karyomegalic tubular epithelial cells in renal biopsy specimens, which differentiates it from other common causes of chronic tubulointerstitial nephritis [3]. Recent studies have linked KIN to mutations in the FAN1 (FANCD2/FANCI-Associated Nuclease 1) gene, which plays a critical role in DNA repair [5, 6, 7]. In this article, we present 4 cases of KIN, each with distinct genetic associations, accompanied by a brief review of the literature. 

## Case 1 

The first case was a 45-year-old male, diagnosed with gout 10 years ago, and renal dysfunction was found in routine examinations. He was admitted to the nephrology clinic with pain in both feet and legs for the last 6 months. He did not have diabetes, hypertension, obesity or any allergies. He had a history of frequent upper respiratory tract infections in his childhood. There was no family member with kidney disease. He did not use alcohol, nephrotoxic drugs, or herbal substances. System findings were natural in physical examination. On admission, the serum creatinine value was 2.71 mg/dL, and estimated glomerular filtration rate (eGFR) value calculated with CKD-EPI was 28 mL/min/1.73m^2^. Liver function tests, hepatitis B, C, human immunodeficiency virus (HIV) serologies, and serum complement levels were normal. In the complete urinalysis, there were no significant casts or cellular elements, hematuria and proteinuria were absent. In 24-hour collected urine, 182 mg proteinuria was detected (Table 1). Renal ultrasound revealed that the location size, parenchymal thickness, and sinus echoes of the kidneys were all within normal limits. 

**Figure 1.**
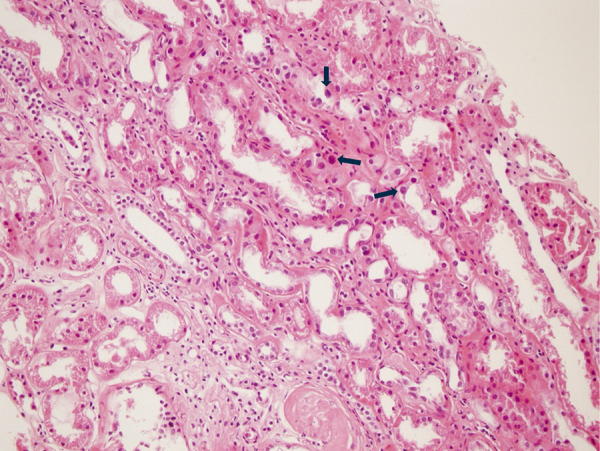
Renal biopsy image of case 1. The arrows highlight karyomegalic cells, characterized by enlarged, hyperchromatic nuclei.

The renal biopsy was performed in 2016 to elucidate the etiology of chronic kidney disease. It revealed the following findings: mild tubular atrophy, nucleomegaly in tubular epithelial nuclei, rare multinucleation and prominence in nuclei. There were no immune deposits present in immunofluorescence microscopy. There was no evidence of immunocomplex nephritis and gouty nephropathy (Figure 1). 

A genetic examination was requested considering KIN in the patient, who fit the picture of chronic interstitial nephritis and had nucleomegaly in the biopsy. In the genetic analysis, we detected heterozygote c.358T>C (p.Cys120Arg) mutation in the UMOD gene by using clinical exome sequencing. According to the Human Gene Mutation Database (HGMD), this variant is assessed as likely pathogenic [8]. Although there was no mutation in the FAN1 gene, pathological diagnosis was consistent with KIN. The patient was started on ACE inhibitor, allopurinol treatment was continued, and he is being followed up with CKD (Table 1). 

## Case 2 

The second case was a 67-year-old female diagnosed with subclinical hyperthyroidism and hyperlipidemia, who had hypertension for 8 years and was referred to the nephrology clinic due to renal dysfunction. Her blood pressure was regulated, she did not have diabetes, obesity, or any allergies. She had been suffering from nocturia for 10 years. There was a history of pharyngitis 4 times a year. There was no history of smoking and alcohol use. She did not use nephrotoxic drugs or herbal substances. There was no history of frequent urinary tract infections. There was no history of kidney disease in her family. She had a history of breast cancer and was undergoing letrozole treatment. Serum creatinine value on admission was 1.58 mg/dL, and the eGFR value was 35.2 mL/min/1.73m^2^. Liver function tests, hepatitis B, C, HIV serologies, and serum complement levels were normal. In the urinalysis, there were no significant casts or cellular elements, hematuria and proteinuria were absent. In 24-hour collected urine analysis, 143 mg/day of proteinuria was detected. No significant findings were detected in renal ultrasonography. 

**Figure 2.**
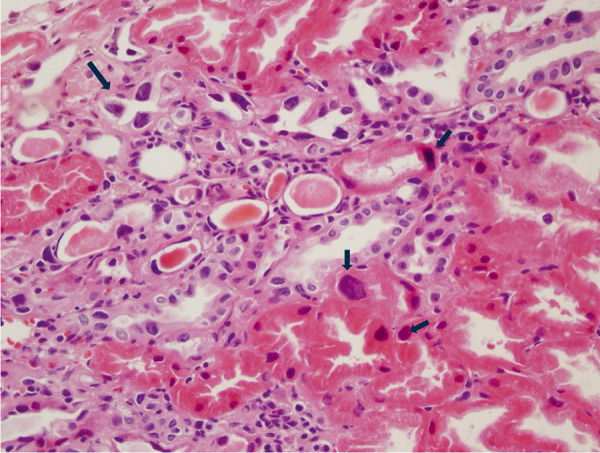
Renal biopsy image of case 2. The arrows highlight karyomegalic cells, characterized by enlarged, hyperchromatic nuclei.

Global sclerosis in glomeruli, nucleomegaly, intranuclear inclusions, and hyperchromasia in tubulus epithelial cells and endothelial cells were detected in the renal biopsy dated 2016, which was performed to determine the etiology of CKD (Figure 2). A pathological diagnosis of KIN was made. The genetic analysis detected c.1972 C>T (p. Arg658Trp) mutation in the FAN1 gene. According to the HGMD guideline, this variant is classified as being of unknown significance. However, the clinical and pathological features of the patient were compatible with KIN, therefore, we concluded that this specific FAN1 mutation was associated with the disease in our case. The patient was started on ACE inhibitor while her existing regimen – including letrozole, levothyroxine sodium, calcium carbonate, metoprolol, acetylsalicylic acid, and atorvastatin – was maintained. The patient last visited our center in 2023. The values from that period are indicated in Table 1. 

## Case 3 

**Figure 3.**
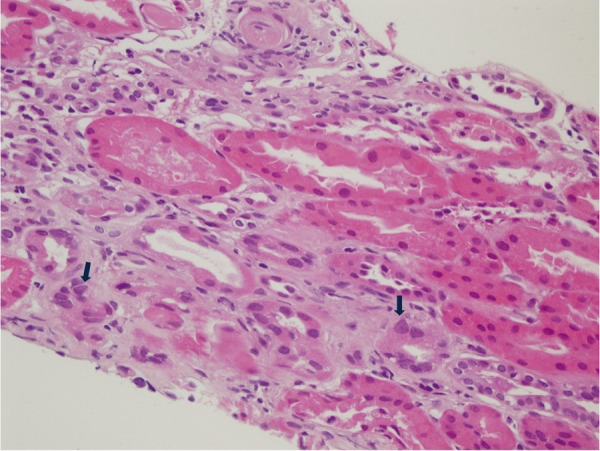
Renal biopsy image of case 3. The arrows highlight karyomegalic cells, characterized by enlarged, hyperchromatic nuclei.

The third case was a 32-year-old female with a history of recurrent nephrolithiasis. During routine investigations, proteinuria was detected. The patient did not have diabetes, hypertension, or any allergies. Her blood pressure was regulated. Her body mass index (BMI) was 29 kg/m^2^. She had a history of bariatric surgery. There was no history of smoking, alcohol, or substance abuse. Physical examination was unremarkable. There was no family history of kidney disease. On admission, the patient’s serum creatinine was 0.79 mg/dL, and eGFR was 104 mL/min/1.73m^2^. 24-hour urine collection revealed 1,443 mg of proteinuria (Table 1). Renal ultrasonography revealed that the location, size, parenchymal thickness, and sinus echoes of the kidneys were all within normal limits. Renal biopsy was performed in 2021 and revealed marked nucleomegaly and hyperchromasia in numerous tubule epithelial cells (Figure 3). IgA deposits were observed on immunofluorescence microscopy. Immunohistochemically, cytomegalovirus and SV40 were negative. Therefore, the patient was diagnosed with IgA nephropathy and KIN. The patient was started on angiotensin converting enzyme inhibitor (ACEi) therapy and is continuing nephrology follow-up. 

## Case 4 

The fourth case was a 45-year-old male. The patient had asthma, psoriasis, and subclinical hypothyroidism. He did not have diabetes, hypertension, or allergies. His BMI was 26 km/m^2^, and his blood pressure was regulated. While being examined for hypothyroidism, elevated creatinine was detected and referred to nephrology. Serum creatinine was 1.76 mg/dL, eGFR was 46 mL/min/1.73m^2^, and rheumatologic markers were negative. 24-hour urine collection revealed 221 mg of proteinuria. The kidney ultrasound showed increased echoes in both kidneys, with a mild increase (grade 1) on the right and a moderate increase (grade 2) on the left. Renal biopsy was performed in 2021 and revealed 9 global sclerotic glomeruli, 2 segmental sclerotic glomeruli out of 26 glomeruli, marked cytomegaly in tubules, marked nuclear karyomegaly in tubule epithelial cells consistent with KIN. Genetic analysis using clinical exome sequencing identified an NM-014967.5 homozygous mutation in the FAN1 gene. 

## Literature review 

**Figure 4.**
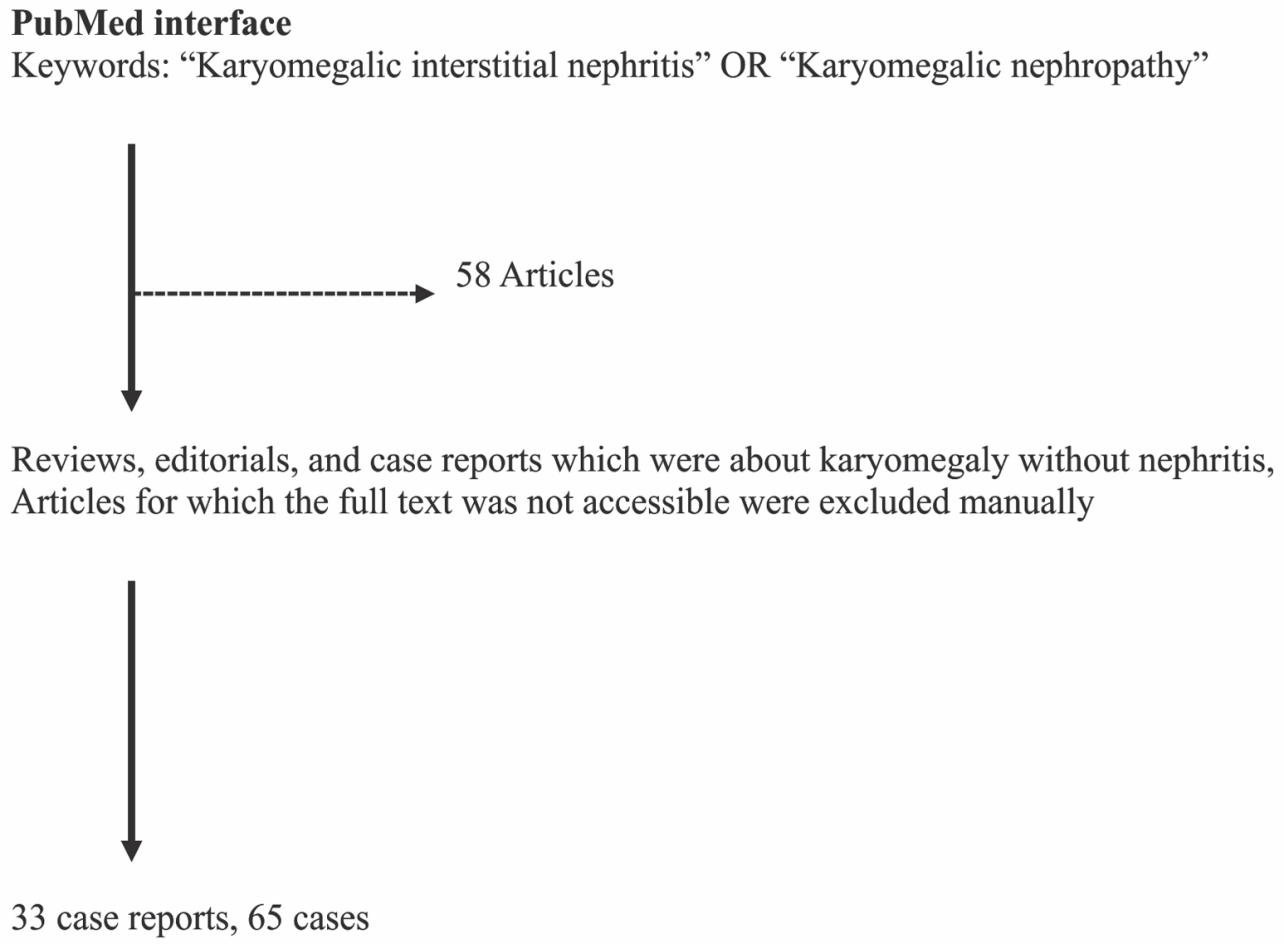
Steps of the literature review.

We used the PubMed interface (pubmed.gov) to make a query using the combination of the following two keyword groups. The first group included the keywords “Karyomegalic interstitial nephritis”, and the second group included “Karyomegalic nephropathy”. Each keyword in the two groups was combined using the logical operator “OR”. We ran the query in August 2024. We excluded reviews, editorials, and case reports that were about karyomegaly without nephritis, alongside 8 articles for which full texts were inaccessible. We found 58 articles in total and manually examined them. After the manual examination and exclusions, we found 33 case reports and case series, and a total of 65 cases about KIN (Figure 4). 

## Discussion 

KIN is a rare yet significant cause of chronic interstitial nephritis, characterized by distinct pathological findings and a multifactorial etiology. In this study, we reviewed 65 cases of KIN, revealing key clinical, genetic, and environmental factors that contribute to its development and progression (Table 2). The patient cohort demonstrated a male predominance, at a ratio of 2 : 1. Patients were aged between 21 and 62 years at diagnosis. Geographically they were distributed across Europe, North Africa, and the Asia-Pacific region, showing that the disease is global. 

Genetic analysis emphasized the critical role of FAN1 mutations in KIN pathogenesis. FAN1 is known for its established function in DNA repair mechanisms [6]. Additionally, a hereditary component is supported by familial clustering and associations with specific HLA haplotypes, such as HLA-A9 and HLA-B35 [3, 5]. Moreover, we identified a potentially pathogenic UMOD mutation in our first case, previously linked to hyperuricemia and uric acid nephropathy, expanding the genetic landscape of KIN. While FAN1 mutations are the most well-established genetic cause of KIN, other genetic variations, including UMOD, may contribute to a similar tubulointerstitial nephritis phenotype, potentially modifying disease expression. This novel finding underscores the necessity of broader genetic screening in patients with CKD of uncertain etiology, facilitating earlier diagnosis and targeted management. The interplay between genetic predisposition and environmental exposures remains a pivotal aspect of disease progression. 

Ochratoxin A, a mycotoxin commonly found in contaminated food, has been linked to renal tubular toxicity and DNA damage, contributing to the development of chronic tubulointerstitial nephritis, including KIN. It is thought to impair DNA repair mechanisms, promote oxidative stress, and induce tubular cell apoptosis, potentially accelerating kidney injury in genetically predisposed individuals. Ochratoxin A exposure, particularly in endemic regions like Tunisia, emerged as a significant environmental risk factor [9]. Furthermore, the association of KIN with prior exposure to nephrotoxic agents, such as alkylating agents like ifosfamide, and exposure to heavy metals, suggests a synergistic effect between genetic susceptibility and external triggers [10, 11, 12]. 

Clinically, KIN presents a spectrum of renal involvement, ranging from asymptomatic proteinuria and hematuria to progressive CKD. Serum creatinine levels at presentation varied widely, reflecting the heterogeneity in disease severity. Notably, more than half of the patients progressed to end-stage renal disease (ESRD), necessitating renal replacement therapy [3, 7, 12, 13, 14, 15, 16, 17]. Systemic karyomegaly has been reported in organs such as the liver, lungs, and peripheral nerves, suggesting a potential link to DNA repair defects, particularly in FAN1-related cases. Extrarenal manifestations were observed in nearly half of the patients, with recurrent respiratory infections and abnormal liver function tests being the most common [3, 4, 7, 9, 12, 13, 15, 18, 19, 20, 21, 22, 23, 24]. Mortality was primarily attributed to advanced disease stages or systemic complications such as septic shock and malignancy [3, 7, 10, 20, 24, 25]. 

Renal biopsy remains indispensable for diagnosing KIN, with the hallmark finding of karyomegalic tubular epithelial cells serving as a critical diagnostic criterion [3]. However, the absence of specific biomarkers for early detection continues to pose a significant challenge. 

Renal transplantation was performed in select ESRD patients, yielding mixed outcomes. While many recipients achieved stable graft function, the recurrence of renal pathology in some cases highlights the need for vigilant post-transplant monitoring and individualized therapeutic approaches [3, 20, 22, 26]. The use of steroids, such as methylprednisolone, was common in managing active inflammation; however, their long-term efficacy remains uncertain, necessitating further investigation [21, 27, 28]. 

The findings of this study reinforce the importance of addressing modifiable risk factors, particularly environmental exposures, to mitigate disease progression. The observation of systemic manifestations in KIN further highlights the need for a multidisciplinary approach to patient care, incorporating nephrologists, geneticists, and environmental health specialists. Management focuses on supportive therapy such as renin angiotensin aldosterone inhibitors, blood pressure control, lifestyle modifications such as dietary adjustments, and regular exercise to support overall kidney health. While steroids have been used in some cases, their long-term efficacy remains uncertain and requires further investigation. Furthermore, patients should be followed up for potential extrarenal manifestations. 

Advances in genomic technologies and long-term follow-up studies are crucial to elucidate the complex interplay between genetic predisposition and environmental triggers. This approach will enable the development of enhanced diagnostic tools and personalized management strategies. 

In conclusion, early recognition, integration of genetic analysis into diagnostic screening, and multidisciplinary approach to managing KIN are pivotal for improving patients’ outcomes. Further research is essential to unravel the underlying mechanisms and address the challenges posed by this rare but impactful condition. 

## Statement of ethics 

The present work was conducted in accordance with the Declaration of Helsinki. 

## Consent to publish 

Informed consent was obtained from the patients for publication of this case series and accompanying images. 

## Acknowledgment 

The first two cases discussed in this manuscript were previously presented as a poster presentation at the European Renal Association (ERA) Congress held in 2021 [29]. 

## Authors’ contributions 

Seyda Gul Ozcan and Nurhan Seyahi conceptualized the study and supervised the project. Durdane Yagmur Ersoy and Ali Osman Polat were involved in data collection. Iclal Gurses contributed to pathological analysis and interpretation. Aysel Kalaycı Yigin assisted in genetic analysis. Sinan Trabulus involved in patient evaluation and reviewed the manuscript critically for important intellectual content. All authors read and approved the final manuscript. 

## Funding 

This research received no specific grant from any funding agency.

## Conflict of interest 

The authors have no conflict of interest to declare. 

**Table 1.** Table 1. Clinical and laboratory parameters of the cases.

**Parameter**	**Case 1**	**Case 2**	**Case 3**	**Case 4**	**Reference values**
Initial laboratory findings					
Serum creatinine (mg/dL)	2.71	1.58	0.79	1.76	0.7 – 1.2
eGFR (mL/min/1.73m^2^, CKD-EPI)	28	35.2	104	46	
Proteinuria (mg/24h)	182	143	1,443	221	< 140
Hematuria	Absent	Absent	Present	Absent	
AST (IU/L)	12	25	18	32	< 40
ALT (IU/L)	16	35	15	32	< 41
Uric acid (mg/dL)	6.2	6.6	6.1	6.8	3.4 – 7
Hgb (g/dL)	11.5	11.4	13.3	11.6	13.6-17.2
Treatment					
RAAS inhibitors	Yes	Yes	Yes	Yes	Yes
Steroids	No	No	No	No	No
Outcome and follow-up					
Last serum creatinine (mg/dL)	3.21	3.3	0.75	2.87	0.7 – 1.2
Last eGFR (mL/min/1.73m^2^, CKD-EPI)	22	14	106	25	
Last proteinuria (mg/24h)	176	173	320	186	< 140
Last hematuria	Absent	Absent	Present	Absent	
Last AST (IU/L)	8	61	14	23	< 40
Last ALT (IU/L)	18	43	11	29	< 41
Uric acid (mg/dL)	6	5.2	5.1	6.9	3.4 – 7
Hgb (g/dL)	12.7	11	12.3	10.5	13.6 – 17.2

eGFR = estimated glomerular filtration rate; CKD-EPI = chronic kidney disease epidemiology collaboration; RAAS = renin-angiotensin-aldosterone system; AST = aspartate aminotransferase; ALT = alanine aminotransferase; Hgb = hemoglobin.

**Table 2.** Review of reported cases of karyomegalic interstitial nephritis.

**Reference **	**First author**	**Year of publication**	**Origin**	**Sex**	**Age at diagnosis**	**Case presentation**	**Serum cr (mg/dL)**	**Karyomegalic cells in kidneys**	**FAN1 mutation**	**Toxin/drug**	**Family history**	**Extrarenal features**	**Outcome**
[5]	Spoendlin	1995	Switzerland	M	27	CKD	2.7	Yes	N/A	No	No	Recurrent upper respiratory tract infections	CKD
[5]	Spoendlin	1995	Switzerland	F	39	Progressive renal failure	3.9	Yes	N/A	No	No	Recurrent upper respiratory tract infections	CKD
[5]	Spoendlin	1995	Spain	F	24	CKD, proteinuria	2.4	Yes	N/A	No	No	No	CKD
[3]	Bhandari	2002	Australia	F	50	Proteinuria and hematuria	2.83	Yes	N/A	No	Yes	No	Ex (post tx ahus, legionella pneumonia)
[3]	Bhandari	2002	Australia	M	21	Impaired renal function after viral illness	2.15	Yes	N/A	N/A	No	Chronic right leg lymphoedema secondary to absent lymphatics, petit mal epilepsy, pneumonia, and congenital fusion of two spinal vertebrae.	N/A
[3]	BhandariS	2002	Australia	M	38	ESRD evaluation		Yes	N/A	N/A	Yes	No	Ex (post tx bronchopneumonia)
[3]	Bhandari	2002	Melanesia	M	29	Proteinuria, microscopic hematuria, creatinine progression	2.26	Yes	N/A	N/A	No	History of childhood asthma, upper-respiratory-tract infection	Lost to follow-up
[3]	Bhandari	2002	Australia	F	9	Asymptomatic microscopic hematuria	N/A	Yes	N/A	N/A	No	No	Lost follow-up
[3]	Bhandari	2002	Australia	F	37	Hematuria, proteinuria, impaired renal function	2.18	Yes	N/A	N/A	Yes	No	ESRD
[9]	Hassen	2004	Tunisia	M	48	Chronic interstitial nephropathy; fever and chills	4	Yes	N/A	Confirmed ochratoxin a exposure	Yes	Hemoptysis, recurrent expectoration	CKD
[9]	Hassen	2004	Tunisia	M	42	Chronic interstitial nephropathy; renal failure	9.2	Yes	N/A	Confirmed ochratoxin a exposure	Yes	Pulmonary fibrosis, chronic bronchitis	CKD
[9]	Hassen	2004	Tunisia	F	32	Chronic interstitial nephropathy discovered incidentally	0.73	Yes	N/A	Confirmed ochratoxin a exposure	Yes	No	CKD
[30]	Palmer	2007	Maori	F	44	Acute respiratory illness, left lower pneumoniae	10.04	Yes	N/A	No	Yes	N/A	N/A
[10]	Mcculloch	2011	United Kingdom	M	15	Ewing’s sarcoma, creatinine progression	4.16	Yes	N/A	Ifosfamide	No	No	Ex, due to tumor recurrence
[10]	Mcculloch	2012	United Kingdom	F	13	Ewing’s sarcoma, renal impairment	1.58	Yes	N/A	Ifosfamide	No	No	CKD
[10]	Mcculloch	2012	United Kingdom	F	18	Metastatic Ewing’s sarcoma, urinary frequency with daytime incontinence	1.5	Yes	N/A	Ifosfamide	No	No	CKD
[31]	Zschiedrich	2013	Germany	M	44	Progressive CKD, mild proteinuria	N/A	N/A	N/A	N/A	N/A	N/A	N/A
[32]	Lucisano	2013	Italy	M	33	Impaired renal function	2.2	Yes	N/A	No	No	No	CKD
[11]	Matsuura	2014	Japan	M	15	Gradual deterioration of renal function, previous osteosarcoma	3.51	Yes	N/A	Previous cisplatin, doxorubicin, high dose-methotrexate and ifosfamide	No	No	CKD
[33]	Radha	2014	Asia	M	8	FSGS, nephrotic syndrome	0.7	Yes	N/A	No	No	No	CKD
[25]	Tagliente	2014	USA	F	33	Progressive dyspnea, post lung transplantation cr increase	1.2	Yes	N/A	No	No	Pneumonia	Ex (liver injury due to post tx antifungal prophylaxis)
[18]	Bennani Guebessi	2015	Morocco	M	22	Chronic renal failure investigation		Yes	N/A	No	No	Recurrent upper respiratory tract infection episodes secondary to bronchiectasis	CKD
[34]	Nakhoul	2015	Israel	F	40	Asymptomatic proteinuria, glomerular hematuria	1.5	Yes	N/A	No	No	No	CKD
[4]	Isnard	2016	Turkey	F	36	CKD	2.3	Yes	Yes	No	No	Mild cholestasis, mild bronchiectasis and interstitial infiltrates of lung bases, history of recurrent respiratory tract infections	CKD
[26]	Ravindran	2019	USA	M	46	Worsening renal function, recurrent kin in the allograft	2.7	Yes	N/A	History of ethanol and drug abuse (cocaine, marijuana, and lysergic acid diethylamide)	No	No	CKD
[12]	Dash	2020	Switzerland	M	58	Progressive renal impairment	2.77	Yes	Yes	Exposed to ionizing radiation	No	Recurrent pulmonary infection, elevated liver enzymes	ESRD; peritoneal dialysis
[19]	Wang	2020	China	M	28	Nausea, AKI, IgA nephropathy	1.64	Yes	Yes	No	No	Multiple polyp-like nodules scattered in the gastric antrum, elevated liver enzymes	CKD
[20]	Murray	2020	Ireland	F	47	Progressive renal failure	6.4	N/A	Yes	No	Yes	Bronchiectasis, elevated liver enzymes	Ex, (2 years after renal tx, malignancy)
[20]	Murray	2020	Ireland	M	44	Deranged renal and liver function	4.64	N/A	Yes	No	Yes	Bronchiectasis, elevated liver enzymes	Ex, (6 years after renal tx, malignancy)
[27]	Koshy	2020	India	F	47	Bilateral pitting edema and mild hypertension	1.52	Yes	Yes	No	Yes	No	CKD
[21]	Law	2020	Pakistan	F	44	CKD	2.52	Yes	Yes	No	Yes	Serum amyloid P scintigraphy showed a moderate amyloid load in the spleen and adrenal glands with equivocal renal uptake,	CKD
[15]	Akyürek	2020	Sweden	F	50	Acute gastroenteritis, elevated creatinine	N/A	Yes	N/A	N/A	N/A	Elevated liver enzymes, bronchiectasis	Renal tx
[7]	Rejeb	2021	Tunisia	M	25	Kidney donor check-up; history of epilepsy and visceral leishmaniasis	2.2	Yes	Yes	N/A	Yes	No	CKD
[7]	Rejeb	2021	Tunisia	M	31	ESRD; treated with peritoneal dialysis	11.3	Yes	Yes	N/A	Yes	Cholestasis	Ex; pulmonary tuberculosis
[7]	Rejeb	2021	Tunisia	M	32	Incidental finding of renal failure after family nephropathy discovery	1.6	Yes	Yes	N/A	Yes	Cytolysis and cholestasis; fibrosis and giant karyomegalic nuclei on biopsy	CKD
[7]	Rejeb	2021	Tunisia	M	39	Discovered during kidney donor check-up	1.8	Yes	Yes	N/A	Yes	Cholestasis	Ex; pulmonary embolism
[7]	Rejeb	2021	Tunisia	M	36	Convulsions and ESRD due to tubulointerstitial nephropathy	10.17	N/A	Yes	N/A	Yes	Cytolysis and cholestasis	Renal tx
[13]	Gueguen	2021	Polynesian	F	31	TIN	N/A	N/A	Yes	No	Yes	Cholestasis, bronchiectasis, interstitial lung disease	ESRD
[13]	Gueguen	2021	Polynesian	F	32	TIN	N/A	Yes	Yes	No	Yes	Cholestasis, bronchiectasis	ESRD
[13]	Gueguen L	2021	Polynesian	M	32	TIN	N/A	Yes	Yes	No	No	Cholestasis, bronchiectasis, lower respiratory tract infections	CKD
[13]	Gueguen	2021	Polynesian	M	39	TIN	N/A	N/A	Yes	No	No	Bronchiectasis	CKD
[13]	Gueguen	2021	Polynesian	M	37	TIN	N/A	Yes	Yes	No	No	Cholestasis, bronchiectasis, interstitial lung disease	ESRD
[13]	Gueguen	2021	Polynesian	M	29	TIN	N/A	Yes	Yes	No	No	Cholestasis, bronchiectasis	ESRD
[13]	Gueguen	2021	Polynesian	M	31	TIN	N/A	Yes	Yes	No	No	Cholestasis, interstitial lung disease	ESRD
[13]	Gueguen	2021	Polynesian	M	18	TIN	N/A	Yes	Yes	No	No	Cholestasis	ESRD
[13]	Gueguen	2021	Polynesian	F	31	TIN	N/A	Yes	Yes	No	No	Cholestasis, lung fibrosis	CKD
[13]	Gueguen	2021	Polynesian	F	56	TIN	N/A		Yes	No	No	Cholestasis	ESRD
[13]	Gueguen	2021	Polynesian	M	41	TIN	N/A	Yes	Yes	No	Yes	Bronchiectasis	CKD
[13]	Gueguen	2021	Polynesian	F	35	TIN	N/A	Yes	Yes	No	Yes	Cholestasis, bronchiectasis, interstitial lung disease	ESRD
[28]	Chand	2021	Asia	M	28	Low potassium, nephrotic syndrome	4.03	Yes	N/A	No	No	No	ESRD; hemodialysis
[28]	Chand	2021	Asia	F	28	FSGS, Evan’s syndrome	7,32	Yes	N/A	No	No	Elevated liver enzymes	Lost to follow-up
[35]	Gupta	2022	India	F	24	CKD [history of hematuria for 6 years)	2.5	Yes	N/A	No	N/A	N/A	Lost to follow-up
[22]	El-Husseiny Moustafa	2023	Egypt	M	37	High creatinine after kidney transplantation	1.2	Yes	N/A	No	Yes	Mildly elevated liver enzymes,	CKD
[23]	Zhu	2023	China	F	32	2 years of deranged renal function	1.36	N/A	Yes	No	Yes	Elevated liver enzymes	CKD
[23]	Zhu	2023	China	F	59	Renal tx in 2014	1.69	N/A	Yes	Exposure of decoration materials	Yes	Recurrent respiratory tract infections	CKD
[23]	Zhu	2023	China	F	51	CKD	1.07	N/A	Yes	No	Yes	N/A	CKD
[36]	Xhamy	2024	Spain	F	59	CKD	1.36	Yes	Yes	No	Yes	Elevated liver enzymes	CKD
[24]	Császár	2024	Hungary	M	44	CKD, pulmonary tuberculosis, renal Tx history	N/A	Yes	Yes	No	Yes	Elevated liver enzymes, karyomegaly observed in peripheral nerve	Ex (sepsis)
[24]	Császár	2024	Hungary	M	47	CKD, mental retardation	N/A	Yes	Yes	No	Yes	Elevated liver enzymes, karyomegaly observed in lung	Ex (cachexia)
[24]	Császár	2024	Hungary	M	42	CKD, type 2 diabetes	N/A	Yes		No	Yes	Elevated liver enzymes	Ex
[24]	Császár	2024	Hungary	M	40	CKD elevated liver enzymes, renal Tx history	N/A	Yes	Yes	No	Yes	Elevated liver enzymes, systemic karyomegaly observed in lung, pancreas	Ex (pulmonary aspergillosis)
[24]	Császár	2024	Hungary	F	39	CKD, peritoneal dialysis with recurrent infections	N/A	Yes	Yes	No	Yes	Elevated liver enzymes, karyomegaly observed in pancreas, portal tracts	Ex (sepsis)
[14]	Leong	2024	USA	F	49	Creatinine progression after receiving brentixumab, Hodgin’s lymphoma	2.75	Yes	Yes	Ifosfamide	N/A	No	ESRD
[16]	Walia	2024	India	F	29	Nausea vomiting and decreased appetite	10	Yes	Yes	No	Yes	No	Renal tx, and asymptomatic since then
[17]	Prema	2024	India	M	36	Sepsis, AKI requiring dialysis	N/A	Yes	Yes	N/A	N/A	N/A	Renal tx, following acute rejection, CKD
	Present case 1	2025	Turkey	M	36	CKD, gout	2.71	Yes	No	No	No	Recurrent upper respiratory tract infections	CKD
	Present case 2	2025	Turkey	F	58	CKD, hypertension	1.58	Yes	Yes	No	No	Recurrent upper respiratory tract infections	Lost to follow-up
	Present case 3	2025	Turkey	F	29	Proteinuria	0.79	Yes	N/A	No	No	No	CKD
	Present case 4	2025	Turkey	M	41	CKD	1.76	Yes	Yes	No	No	Asthma	CKD

F = female; M = male; CKD = chronic kidney disease; ESRD = end-stage renal disease; Ex = exitus; Serum cr = serum creatinine; **N/A = not available**; TIN = tubulointerstitial nephritis; tx = transplantation; FSGS= focal segmental glomerulosclerosis; AKI= acute kidney injury.
